# Relative Accuracy Evaluation

**DOI:** 10.1371/journal.pone.0103853

**Published:** 2014-08-18

**Authors:** Yan Zhang, Hongzhi Wang, Zhongsheng Yang, Jianzhong Li

**Affiliations:** Department of Computer Science and Technology, Harbin Institute of Technology, Harbin, China; Center of nonlinear, China

## Abstract

The quality of data plays an important role in business analysis and decision making, and data accuracy is an important aspect in data quality. Thus one necessary task for data quality management is to evaluate the accuracy of the data. And in order to solve the problem that the accuracy of the whole data set is low while a useful part may be high, it is also necessary to evaluate the accuracy of the query results, called relative accuracy. However, as far as we know, neither measure nor effective methods for the accuracy evaluation methods are proposed. Motivated by this, for relative accuracy evaluation, we propose a systematic method. We design a relative accuracy evaluation framework for relational databases based on a new metric to measure the accuracy using statistics. We apply the methods to evaluate the precision and recall of basic queries, which show the result's relative accuracy. We also propose the method to handle data update and to improve accuracy evaluation using functional dependencies. Extensive experimental results show the effectiveness and efficiency of our proposed framework and algorithms.

## Introduction

Data quality problem plays an important role in business analysis and decision making [1]–[4], and has been studied in different areas, such as statistics, management science, and computer science [5]. Dirty data is a major reason for data quality problem. Many surveys reveal that dirty data exists in most database systems. For example, a survey [6] reports that over 65% of the inventory records at retailer Gamma were inaccurate at the store-SKU level. The consequences of dirty data may be severe. Dirty data with uncertainty, duplication or inconsistency may leads to ineffective marketing, operational inefficiencies, inferior customer relationship management, and poor business decisions. For example, it is reported [7] that dirty data in retail databases alone costs US consumers $2.5 billion a year. Hence it is extremely urgent to estimate data's quality before they are used.

Data quality has many aspects including accuracy, inconsistency, concurrency and completeness. Among them, accuracy is an important one. Accuracy is defined as the closeness degree between the measurements of a value and corresponding actual (true) value. In many applications, inaccurate data will mislead the decision. To make sure the usage of data, the accuracy of data should be estimated before they are used. Our preliminary work studies the accuracy evaluation on the whole data set [8], which is called absolute accuracy.

A case is that the accuracy of the whole data set is low but that of a share containing the query results may be high, so it is necessary to evaluate the query result's accuracy which is called relative accuracy. For example, we have a database which collects the sensors' data. After some time, a sensor gets wrong, so the quality of such database becomes low. But if we want to query some data with timestamp before the time that the sensor gets wrong, the database could return results with high quality. With such cases, it is important to evaluate the accuracy of the query and query result.

Another example application that will be benefit from our method is metaknowledge [9]–[12], large corpora of written text, both scientific and literature – which is becoming increasingly available in digitized form. The accuracy estimation could be used to evaluate the quality of metaknowledge and further evaluate its usability.

With its importance, the estimation of relative accuracy brings following technical challenges.

- The data may be from different data sources and in different data model with different accuracy, including structured data model, semi-structured data model and even unstructured data model. The relative accuracy evaluation method should be adapted to all these models.
- Among the data set, different values may refer to the same real-world entity, and we need to estimate the true value of the entity attribute if the entity does not have theexplicit one.
- There are many different types of data. For different types, different estimation approached method should be applied.
- There are many types of queries.Query analysis needs to be executed and the precision and recall of the query results needs to be evaluated.

Current work seldom considers the evaluation of accuracy with different data types. Only our preliminary work [8] proposes evaluation method for absolute accuracy [13]. considers accuracy estimation. However, in that paper, only the category type is considered. And also for a value, in their system, this value can only be considered as true value or false value. But actually in real applications, there are many other data types. For example, in sensor network, the true value is 1.0. For two data sensors A and B, the measured result of A is 2.0 and that of B is 1.5. Clearly, the accuracy of B is better than that of A. In such case, the accuracies of A and B cannot be distinguished even though they are different.

Even though with true value estimation methods [14]–[16], mean squared error (MSE), which is the expected value of the squared error loss or quadratic loss. MSE measures the average of the squares of the “errors”., can be used to estimate the accuracy directly. However, the truth discovery methods are not related to accuracy and are not suitable for the accuracy estimation. And different data types also have different evaluation method. In order to unify the accuracy measurement metric of different data types, we define a new accuracy metric ARE(average relative error) which is based on the mean value of data values' relative error.

To evaluation ARE, we propose a relative accuracy evaluation framework for relational database with different data types, which could also be extended to other data model. This paper makes the following contributions.

1. We propose a general accuracy evaluation framework mainly for relational database with different data types, which could also be extended to other data models.
2. According to the differences in evaluation method for data in various types, we classify the data types into three classes.
3. We propose efficient accuracy evaluation algorithms for three data types in two cases of in presence and absence of true values.
4. We design the strategy to handle data update and the method to use the functional dependency to improve accuracy evaluation.
5. We propose the methods to evaluate the precision and recall of the basic query operations and to evaluate the overall accuracy of the query results, which will be combined to compute the relative accuracy of the query.

In the following parts, we first introduce the framework of the relative accuracy evaluation. As our framework is based on the accuracy of the attributes, we develop attribute accuracy evaluation algorithms for each category in cases of in presence and absence of true values, and show how our framework works at these situations. We also propose the strategy to handle data updating and to use functional dependency to improve accuracy evaluation.

The rest part of this paper is organized as followings. Section 2 proposes the basic framework of relative accuracy evaluation. Section 3 and Section 4 discuss the evaluation algorithms in presence and absence of true values, respectively. Section 5 gives the method to handle data updating and the strategy to improve accuracy evaluation using functional dependencies. The experimental results and analysis are shown in Section 6. Section 7 discusses the related work and section 8 draws the conclusions.

## Framework

As we know, a relational database consists of relational tables, a relational table consists of tuples, and a tuple consists of different attributes. Therefore, we use the accuracy of attributes to evaluate tuples' accuracy, use the accuracy of tuples to evaluate the table's accuracy, and use the accuracy of tables to evaluate the database's accuracy. As a result, we convert the problem to evaluate the accuracy of the attributes [8]. This strategy also could be extended to other data models. The evaluation of the accuracy of a data object can be a combination of the evaluation of its attributes' accuracy.

Using attributes as the basic unit of evaluation does not mean the neglect of the relationships between the attributes. We note that latent relationships among the attributes will affect the accuracy evaluation. It is defined as entity relationship. It means that different attribute value mays describe the same attribute of a real-world entity. With entity relationships, during the evaluation, some attributes with different values may share the same true value. We would use this character as a base to compute the accuracy of the measured data, since if all the measured data are independent, we could not compute the error distribution without enough priori knowledge. Other attribute types are similar.

With above discussions, our evaluation methods will take attributes as basic units and consider the relationship among them. In this section, we propose an overview of the evaluation framework. At first, we show the framework of the relative accuracy evaluation; and then the attributes are classified according to the different accuracy evaluation methods, which would be used as the first step of framework; at last, we describe the methods to compute the rough accuracy of the basic query operations. Such accuracy could be used to define and deduce other operations and this would give users the first impression about the query.

### 2.1 Accuracy Evaluation Framework

The framework of the relative accuracy evaluation includes four phrases.

1. The types of attributes are classified according to the evaluation methods of attributes.
2. The accuracy for each type of attributes is evaluated.
3. The rough accuracy of the query is computed and users would decide whether the query is suitable to be executed.
4. The precision, recall, F-measure of query and the absolute accuracy of the query's results are computed, which are combined to show the relative accuracy of queries.

The first phrase is performed according to data format and data semantics [8]. For example, for numerical value including integral numbers and floating numbers, it is obvious that it belongs to the measurable type; string data and set data belong to the comparable data type, and gender and level data belong to the category data.

In the second phrase, we use statistics theory to compute the accuracy of attribute. As different data types have different dimension, in order to unify the accuracy measurement metric of different data types, we define a new accuracy metric which use the mean value of data values' relative error to represent the data's accuracy. We use it as the accuracy measure for values in the same attribute. The details of this phrase will be described in Section 3 and Section 4 for the cases of presence and absence of true values, respectively.

In the third phrase, we first give the rough accuracy of query using the accuracy of attributes based on the probability analysis. This step will give users the first impression about the query, and this is performed offline which will be very efficient though it maybe not so accurate.

In the fourth phrase, we compute the precision, recall, F-measure of the query and the absolute accuracy of the query's results. The precision of a source *s* is the probability of its positive claims being correct; the sensitivity or recall of a source *s* is the probability of true facts being claimed as true. A measure that combines precision and recall is the harmonic mean of precision and recall, the traditional F-measure is as follows.
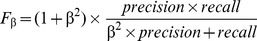
(1)We use *β* as our evaluation criteria to describe the relative importance between recall and precision. A special case is *β* = 1, where recall and precision are evenly weighted.For absolute accuracy of a data set, we use the average of ARE of different types of attributes to represent it. It is denoted as follows.
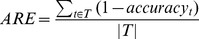
(2)where *T* is the set of attributes, and *accuracy_t_* is the accuracy of attribute *t*. Since the quality of the query is not only related to the accuracy of query attributes and but also the global accuracy of the results, in order to obtain the relative accuracy of the query, we need to consider both of them. Therefore, we use the following quadruple to represent the relative accuracy of the query results.
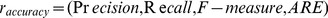
(3)where Precision, Recall and F-measure are the precision, recall and F-measure of the result, and ARE is the accuracy of the result set.

### 2.2Absolute Accuracy Evaluation

Since the attributes may be in various categories, although the semantics of accuracy on them are the same, the accuracy computation methods of them are different. According to their difference, the attributes are classified into three types [8].

- **Measurable Attribute:** The values of such attribute are continues and can be modeled as some distribution. Such attributes include the values from the measure instruments, such as temperature and humidity.
- **Comparable Attribute:** The values of such attribute are not continues and no distribution can be derived from the values. However, the difference of such values can be computed. That is, the distance between the input value and true value can be computed. For example, both the name attribute and some set attribute belong to this type.
- **Category Attribute:** The difference between two values of such attribute cannot be computed. The difference of such attribute can only have a rank instead of concrete value. For example, the gender attribute and the rank attribute.

As different data types have different dimension, the accuracy metric is proposed as well as the accuracy evaluation method of a given data set according to the data type. We will introduce the metrics and evaluation methods in Section 3 and Section 4.

### 2.3 Query analysis and the Probability Calculation

The quality of the query results is related to the accuracy of query attributes and the overall accuracy of the results, in order to obtain the relative accuracy of the query, we need to consider both of them. We will first introduce the query analysis and its rough accuracy calculation approaches.

The operations of queries are varied, such as selection, projection, join, division, union, difference, intersection and Cartesian. They can be defined and derived by five basic operations, selection, projection, union, difference and Cartesian product. The following is the analysis and rough accuracy evaluation of five base operations.

#### 2.3.1 Selection

The selection is also known as the restriction. It selects tuples from database which have to satisfy the given conditions, denoted as *σ_F_*(*R*) = {*t*|*t*∈*R*∧*F*(*t*) = ‘true’}, where *F* represents the selection criteria. *F* is a logical expression, which takes a logical value of true or false. The basic form of *F* is *XθY*, where *θ* represents a comparison operator, and it can be >, ≧, <, ≦,  =  or <>. And *X* or *Y* may represents an attribute name, a constant or as a simple function. We can further carry out logic operations on these basic selection criteria, such as non (¬), and (∧), or (V). The probability calculation is based on the accuracy of the attribute. If X or Y is a constant, then we can only use the accuracy of attributes which are used to represent the query accuracy; if both of X and Y are attributes, we use their accuracy's production to represent the query probability, that is *P_F_*_(*t*)_ = *P_X_*×*P_Y_*; the corresponding probability formula for ¬ is *P*_¬*A*_ = 1−*P_A_*; for ∧, it is *P_A_*_∧*B*_ = *P_A_*×*P_B_*; for V, it is *P_A_*_V*B*_ = *P_A_*+*P_B_*−*P_A_*×*P_B_*.

#### 2.3.2 Projection

The projection on the relation R is to select some particular attributes to form a new relation from R. It is denoted as *π_A_*(*R*) = {*t*(*A*)|*t*∈*R*}, where A is a set of attributes from R. We need not to compute its accuracy, as it will select the entire column. We can use the mean value of the accuracy of selected attributes to represent the rough projection accuracy. For example, if the selected attributes are *A* and *B*, then the rough projection accuracy = (accuracy(A)+(accuracy(B))/2.

#### 2.3.3 Union

The union of relation R and the relation S is denoted as *R*U*S* = {*t*|*t*∈*R*V*t*∈*S*}, where R and S share the same attributes. As the relation union will only remove the tuples belonging to both two relations, we use the formula *P_R_*_U*S*_ = 1−*P_R_*×*P_S_* to represent the rough probability of union.

#### 2.3.4 Difference

The difference of relation R and the relation S is denoted as *R*−*S* = {*t*|*t*∈*R*∧*t*∉*S*}, where R and S share the same attribute. As in the difference, the dataset R will only removes the tuples belonging to the second set. We use the formula *P_R_*_−*S*_ = *P_R_*×(1−*P_S_*) to represent the rough probability of difference.

#### 2.3.5 Cartesian Product

The Cartesian product considered here is exactly the extended Cartesian product, since the unit is tuple. The Cartesian product of relation R with *m* attributes and relation S with *n* attributes is a relation containing 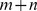 attributes. It is denoted as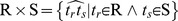, and it is generally not used directly, but as the basic of join and other operations. We use the formula *P_R_*_×*S*_ = *P_R_*×*P_S_* to represent the rough probability of Cartesian product. However, if the Cartesian product only uses a portion of relations just like equijoins and natural join, we could only use the product of the accuracy of attributes which are used to represent the rough probability of Cartesian product.

**Example 2.1** The join could combine by selection and Cartesian product, and is also called 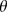 join, which is to select some tuples satisfy certain conditions from the Cartesian product of two relations. It is denoted as 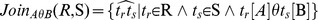, where *A* and *B* represent some comparable attributes from R and S and 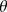 is the comparison operator. Two of the most important and also the most common join are equijoin and natural join. The 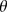 of the equijoin is “ = ”,which means selected tuples which have equal attribute values at the attribute A and B from the Cartesian product of two relation, denoted as 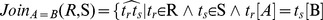; the natural join is a special equijoin, which request not only the equal attribute value but also the same attribute type, denoted as 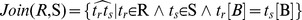. We can use the formula 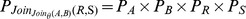 to represent its rough query result probability.

In this paper, to simplify the discussion, we treat that all data objects and types share the same importance. The accuracy in case that data objects or types have different importance could be evaluated by adding weights on each item in the accuracy evaluation formula.

In Section 3 and 4, we will propose attribute accuracy evaluation algorithms for data type in each category in cases of in presence and absence of true values, and show how our framework works at these situations.

## Accuracy Estimation with the True Values

Accuracy is defined as the closeness degree between the measurements of a quantity and the quantity's actual (true) value.As different data types have different dimension, we need a metric to measure the accuracy of different data types. We first propose a new metric to uniform describe the accuracy of different data types and then describe how to evaluate the accuracy at the ideal situation in which the attributes have true values.

In statistics theory, mean squared error (MSE) is often used to estimate the accuracy of observations. However, different data types have different dimension, in order to unify the accuracy evaluation metric of different data types, we define a new standard ARE(average relative error) which use the mean value of data values' relative error to represent the data's accuracy. The relative error of a parameter θ is denoted as: 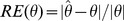, where θ is true value of a parameter and the 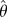 is the observation of θ. And the ARE of attribute is denoted as follows
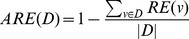
(4)Where *D* is the set of the attribute values, *v* is a value which belong to *D* and RE(*v*) is the relative error of *v*. In the remaining part of this paper, we also use D to denote the set of attribute values.

In presence of true values, the computation of *ARE* looks trivial. However, for different data types, the computation of ARE is different. We will discuss the evaluation methods for different data types with true values, respectively.

In this section, the evaluation methods involve true values. In order to distinguish true values from the values of attributes in the data set which possibly contain inaccuracy or even false values, in the remaining part of this paper, we use observations to refer the value of attributes in data set.

**Measurable Attribute:** For measurable attributes, the accuracy for a set of observations is computed as followings.
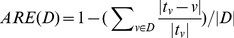
(5)


Where 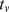 is the true value of *v*. With true value, the ARE is computed as the average of the relative accuracy between the observations and the true value.

**Comparable Attribute:** For comparable attributes, we define the distance function first, and the accuracy evaluation of comparable type is computed as following.
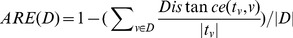
(6)where 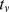 is true value relative to observation value 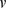 and |*t_v_*| is the length of *t_v_*. *Distance* is a distance function defined on the comparable data type, for example, it can be edit distance for string data, or Jaccard distance for set data type.

**Category Attribute:** For category attributes, the difference between values cannot be computed as before. Thus, the ARE is computed as the expectation of the observation equaling to the true value. It is denoted as
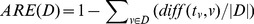
(7)where for the function *diff*() is computed from, if *t_v_* = *v*, it returns 0; others, it is computed according to the rank of the difference between *t_v_* and *v*.To computer *diff*(), we model the values in a category attribute as a graph G = (*V*, *E*), where *V* is the set of all values and each (*u*,*v*)∈*E* represents that *v* is the most similar to *u* among all values in *V*. Then diff(*u*,*v*) is defined as the length of the shortest path in *G*.

**Precision and recall:** Theprecision and recall of a query can be computed according to the definition. We denoted the case that the observation is true and the fact is true as 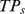, that the observation is false but the fact is true as 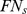, that the observation is true but the fact is false as 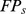, andthe case that the observation is false and the fact is false as *TN_S_*. The precision of a query is denoted as *precision* = *TPS*/(*TP_S_*+*FP_S_*), and the recall is denoted as *recall* = *TP_S_*/(*TP_S_*+*FN_S_*). With true values, they are easy to compute.

## Accuracy Estimation without True Values

In many cases, the true value for an attribute is unknown. In this case, the accuracy computation is more difficult and the true values need to been estimated with existing observations. Based on the observations, we estimate the accuracy without true values for different data types.

### 4.1 Measurable Data Type

For measurable data type, we noted that if all the data are independent to each other, it is impossible to compute the true values and get the accuracy of the data without enough priori knowledge. Since we could not often obtain enough priori knowledge and many tuples may describe the same entity, we could use the entity resolution technology [17] to find tuples which describe the same entity. Then we obtain a series of measurable data which share the same true values. We first compute the ARE of every entity, and then use them to compute the ARE of the whole data set.

Generally, in a certain sample volume, the metric which evaluate the quality of point estimation is always the distance function which measures the distance between the point estimate value 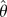 and the true parameter value*θ*. The most commonly used function is the square of the distance, and because of the randomness, we can compute the expectation of the function. The mean square error 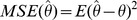 is the most general metric of point estimation. And naturally, we wish to estimate the MSE as small as possible.
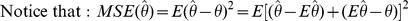
(8)

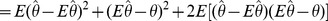
(9)

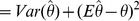
(10)As we can see, the MSE is composed by two parts which are the variance of the point estimation and the square of the deviation. In the case of the certain sample volume, the variance of 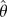 is certain. As long as 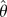 is an unbiased estimation of *θ*, we can minimize the MSE 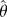. As we know, in a series of observation values, the mean value is an unbiased estimation of the true value, so we can use the mean value to represent the true value to minimize the MSE.

Under the case of minimizing the MSE, we use the mean value to compute the ARE of each entity. As a result, we can get the following formula.
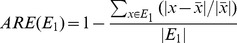
(11)where 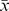 is the average value of all *x* in *E*_1_.
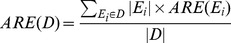
(12)


### 4.2 Comparable Type

Unlike the measurable type, it is hard to find true value for comparable type. As it is alsohard to define the mean value of the attribute of an entity, we define a new measure to find the most-liketrue values as followings.
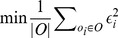
(13)

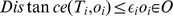
where the function *Distance* is to measure the distance between the observation *o_i_* and its true value *T_i_*, and *ε_i_* is a variable representing the relative error for observation *o_i_*. *Distance* can be the edit distance for strings or Jaccard similarity for sets.

As far as we can see, the most possible true value of one entity's attribute is one of values which describes that entity, but also maybe do not appear with very small probability. It is almost impossible to obtain the true value without enough prior knowledge, if it does not appear. We usually could not have enough prior knowledge in the real world, so we choose the true value from the observation values. Maybe, we could not get the true value, but it is a really small probability event.

We denote different observations as *O* = {*o_1_*,*o_2_*,…,*o_n_*} and the true value as *t*. We use the follow metric to choose the possible true value from the observation values.
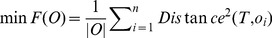
(14)where *T* is the selected true value of all observations *o_i_*.

By enumerating every unique observation value, we could get the most possible true value which minimize *F*(*O*). Though the value 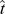 is the biased estimation of the true value, it can minimize the distance function.

Using the estimation value 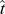, we get the following formula:
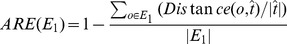
(15)

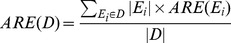
(16)


Our evaluation method could alsoachieve*O*(n) time complexity with the entity resolution technology which using the hash method.

### 4.3 Category Type

#### 4.3.1 Model

For category types, we also utilize the entity resolution technology. We denote each entity as *e* and the set of entity as E. We assume that the tuples share the same model, which belong to one same entity. We denote the possible true values of the entity as *T*. Since for a category attribute, the only information for the true value is from the observations. It means that without external knowledge, the true value should be one of the observations. The parameters of the model are defined as following:θ = {μ;r}, where *μ_i_* represents the probability of the true value is *t_i_*, r represents the error transition matrix which is a |t|×|t| matrix and its element r_12_ represents the probability of the observed value is t_2_ in the case of the true value is t_1_. The accuracy of one entity is defined as 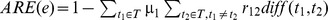. Therefore, we compute the global accuracy as 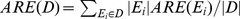.

#### 4.3.2 Solutions

Based on the model, we attempt to use EM algorithm [20] to estimate the parameters of the model. The observable variable of model denoted as *O*, the latent variable denoted as *T*, the parameter denoted as *θ*. The likelihood function of the observable variable denoted as following: 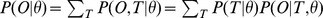. The goal is to compute the maximal likelihood estimation for *θ*.

Now, we design EM algorithm to solve this problem. At first, *θ* ^(0)^ is initialized by this way: *μ_t_* is initialized by choosing a random value from range (0,1), and it is need to make sure 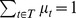; 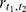 is initialized by choosing a random value from the range of (0,1), and it needs to make sure 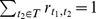. And *diff*(*t*,*o*) is defined according to Section 3.

At the E step, θ^(i)^ denotes the *i*th iteration value of the estimation value*θ*. In the next step, the following formula needs to compute.
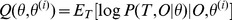
(17)

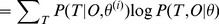
(18)


For a specific true value *t*, 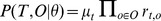.
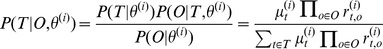
(19)


Because for a specific true value *t*, 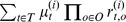 can be seen as a constant, so it can be neglected as our goal is the evaluation value of θ when to maximize *Q*(*θ*,*θ* ^(i)^). Finally, we get
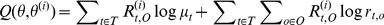
(20)where 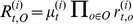.

In the M step, the estimation of *θ* ^(i+1)^ for the *i*+1th iteration is computed as the *θ* to maximize *Q*(*θ*,*θ* ^(i)^). Then E step and M step are repeated until coverage.

With the condition of 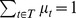 and 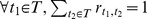, the problem of computation optimal θ is converted to the following optimization problem.
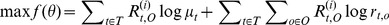
(21)

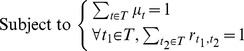



It is supposed that *T* = {*t_1_*,*t_2_*,…,*t_k_*}.Using Lagrange duality and Lagrange multiplier, we get the Lagrange function as following.
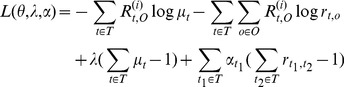
(22)


Setting the gradient 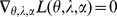 yields the system of equations as following.
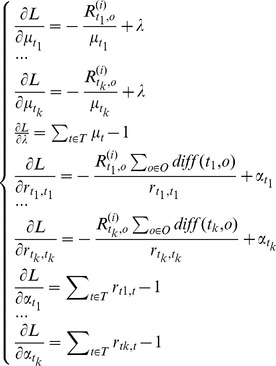
(23)


We can get the solution of equations as following:
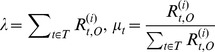
(24)

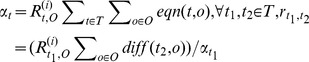
(25)


**Example 4.1:** Suppose that an observation set is {A, A, A, A, B, B, C, C}, we could get the parameters of the model as *θ* = {*μ*;*r*}, where *μ* = {*μ_A_*,*μ_B_*,*μ_C_*} and *r* = {*r_AA_*,*r_AB_*,*r_AC_*;*r_BA_*,*r_BB_*,*r_BC_*;*r_CA_*,*r_CB_*,*r_CC_*}. The parameter μ is initialized as {0.5, 0.25, 0.25} and 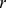 is initialized as {0.6, 0.2, 0.2; 0.3, 0.4, 0.3; 0.4, 0.2, 0.4}. Then, we could use the formula (24) and (25) to iterate until the parameters convergence. At last, we could get the accuracy of the entity.

### 4.4 Implementation

In this subsection, we introduce the implementation issues for the evaluation methods.

**Accuracy Evaluation for Measurable Data Type:** To implement such evaluation, we perform entity resolution with hashing [17] at first. Then ARE is computed for each entity according to Eq. (11). At last, the global ARE is computed based on Eq. (12). Thus, our evaluation method could get *O*(n) time complexity.

**Accuracy Evaluation for Comparable Data Type:** To implement the evaluation for measurable attribute *a*, we also perform entity resolution on the data [17] at first. Then, for each entity *e* with all possible values *O* = {*o*_1_,*o*_2_,…*o*_n_} in the attribute *a*, we enumerate each *o_j_*∈*S* in as the true value and compute 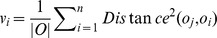 according to Eq. (14). After that, the *o_i_* leading to the minimal vi is selected as 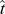 and ARE for e is computed according to Eq. (15). At last, ARE of the global dataset is computed according to Eq. (16).

**Accuracy Evaluation for Category Data Type:** According to Section 4.3,the evaluation is accomplished with EM algorithm. As the framework of EM algorithm, random values are assigned to parameters *μ* and *r*. Then*μ* and *r* keep on updating iteratively according to Eq. (24) and Eq. (25) until convergence. After convergence, with *μ* and *r*, the accuracy of a single entity *e* is computed as 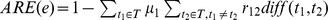 and then the global accuracy is computed as 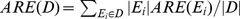.

### 4.5 Precision and Recall without true values

Without true values, the precision and recall of the query is difficult to compute. In order to get the accuracy of query which represents how close it is to the real situation, we would use the truth to find methods discussed above to evaluate the precision and recall of the query results.

For measurable attribute types, we use the mean value 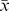 of the values which share the same true value to represent the true value; for comparable attribute type, we use the value 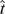 which could minimize the function *F*(*O*) denoted as formula(14)to represent the true value. For category attribute types, using the model in Section 4.3.1, we use the value *t* with the largest μ_t_ to evaluate the true value. For category attribute type, we could also use maximum likelihood estimation to find the true value, just as we use the value account for the largest proportion of all the values which share the true values to represent the true value We could also use the proposed relative accuracy computation method to assign the tuple attributes weight factor to determine the true value for category attribute type.

With the evaluated true value, we can use formula TP_S_/(TP_S_+FP_S_) and TP_S_/(TP_S_+FN_S_) proposed in Section 3 to compute the precision, recall and F-measure of the query.

Our framework could also handle the dynamic data updating, we will talk about it in the next section, as well as how to improve accuracy evaluation using the relationships between the attributes.

## Data Update and Functional Dependency

In Section 3 and Section 4, we propose the accuracy estimation methods. It assumes that the data set is static, but actually the data set always changes. In this section, we discuss how to handle data updating. As we find that the relationships between the attributes could be used to improve the accuracy evaluation, we will also discuss it in this section.

### 5.1 Data update

In order to adapt our framework to data updating and avoid recomputing the accuracy using the whole data set, we need to consider methods to handle data update. In order to facilitate calculation,we need to record the computed data. Two kinds of information need to record, one is the accuracy of attributes, and the other is the entity relationship between the tuples, which means some tuples referring to the same real-world entity.

There are three kinds of data updating operations, data modification, tuple insertion and tuple deletion. For data update, we denote the entity before modified as*E* and that after modified as 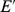. We denote their accuracies as*ARE*(*E*) and *ARE*(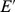), respectively. The data attribute's accuracy before modified is denoted by*ARE*(*T*). We denote the data set as *D*. Since data modification does not change the size of the data set, we propose following formula to update the accuracy of attribute.
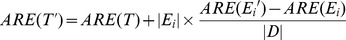
(26)


For tuple insertion and tuple deletion, we denote the size of data set after operation as 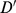. Then we propose the following formula for accuracy updating.
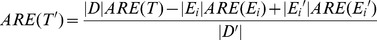
(27)


From formula (26) and (27), we can see that, if the size of data set is very large and the accuracy change is small, we need not to update the accuracy of attribute timely. We can update the accuracy after the number of change up to a constant number, which can be set manually. It can facilitate the relative accuracy evaluation algorithm.

### 5.2 Improving accuracy evaluation using functional dependency

When we defined the schema of relational database, we usually have functional dependencies between attributes. The functional dependency is defined as follows. Given a relation *S* with attributes set *U*(*B*_1_,*B*_2_,…,*B*_n_), *X*, *Y* are subsets of *U*. For any two tuples of *S*, if u[*X*] = v[*X*], then we can get u[*Y*] = v[*Y*]. We called this as *Y* functional dependence by *X*, denoted as *X*→*Y*. We can change the query plan using the functional dependency. For example, if *X*→*Y*, the query on attribute *Y* could convert attribute *X*. From this point, we propose the method to accelerate accuracy evaluation.

#### 5.2.1 Accuracy range for global accuracy of data set

As functional dependency exists in most databases and the query plan could be replanned and executed using only a part of attributes set. We can use a small attributes set to represent the whole attributes. Based on this point, we can use the accuracy of partial attributes' to represent the accuracy of the whole data set.

We attempt to use functional dependencies between attributes to discover more information between attributes, and mainly to find candidate keys. As we know, 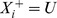 from the knowledge of functional dependencies and closure, so we can represent the whole tuple using the candidate keys. For a query on an ordinary attribute, we can get the new query plan by functional dependencies and query rewriting. Hence we can determine the accuracy of a dataset using the accuracy of candidate keys.

Candidate keys discovery algorithms have been studied in [18] [19] and are not the focus of this paper. With candidate keys, we can filter out some attributes with low accuracy but can be deduced by candidate keys. This can make great improve on accuracy evaluation of data set.

Suppose a table have two attributes, A and B, and attribute B depends on attribute A. All queries about attribute B can be transformed into a query on attribute A, and we can get the upper and lower bounds of the table's accuracy according to the accuracy of A and B. If *ARE*(*A*)>*ARE*(*B*), the accuracy of the table belong to the range (*ARE*(*B*),*ARE*(*A*)); if *ARE*(*A*)<*ARE*(*B*), the accuracy of the table is in range (*ARE*(*A*),*ARE*(*B*)).

Usually, there are more than one candidate keys in the relational schema. Assuming that the set of candidate keys is {X_1_,X_2_,…,X_n_}, our strategy is as follows. We first sort the attributes of relation according to their accuracy computed before. We then find the attributes which are not candidate keys but their accuracy is higher than the minimum accuracy of candidate keys. They form the set 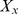, and we can get the range which the accuracy of relation belong to. That is,
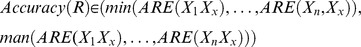



We use an example to illustrate the strategy.

**Example 5.1:** The relational schema is R(A,B,C,D,E) and its functional dependencies are Y = {A→BC, CD→E, B→D, E→A}.By candidate keys discovery algorithm, we can get the following candidate keys:
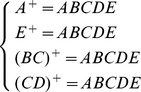
As shown above, we know the candidate keys of R are A, E, BC, CD. Compute {*ARE*(*A*), *ARE*(*B*), *ARE*(*BC*), *ARE*(*CD*)}, denoted 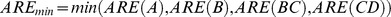 and 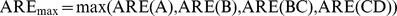, we can get the accuracy range of R as 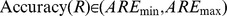.

#### 5.2.2 Suggestions for improving query accuracy

As a query plan could be reenacted using functional dependencies, so we can use it to improve the relative accuracy of queries.

If we can find the mapping relation between attributes using functional dependency, then we can apply this to improve the relative accuracy of query. For example, suppose each place name corresponds with only one encoding, denoted encoding as attribute *X* and place name as attribute *Y*,then the mapping could be denoted as *X*→*Y*. If the accuracy of attributes has already been computed and marked, when a query is on attribute *Y*, if the accuracy of *Y* is higher than *X*, then we can execute query on *Y* directly; if the accuracy of *X* is higher than *Y*, then we can execute on *X* by mapping rules. For mapping rule making process, we can collect all the values when *X* and *Y* appear concurrently to make mapping rule or table. In addition, we can also use the closure of schema to find all the functional dependencies. Through attributes' accuracy record and the functional dependency between attributes, we can reenact the query plan, thereby increasing the relative accuracy of the query.

## Experimental Results

In order to evaluate the performance and efficiency of the relative accuracy evaluation, we carried out a series of experiments. In this section, we describe the process through which we obtained the test data. Hereafter, we carry out extensive experiments on basic queries and analyze their results. To the best of our knowledge there are no publicly available systems which directly evaluate the relative accuracy of queries and the global accuracy of query results. Most of the query estimation algorithms focus on how to produce the high quality results relative to query condition, but they do not generally involve the global accuracy of the result set. We do not only care about the accuracy of query, but also the accuracy of the query results. Our experiments are conducted on a 3 GHz Inter(R) Core(TM) 2 Duo CPU with 4 GB main memory.

### 6.1 Test data

Since there is no benchmark dataset available for evaluating the performance of our accuracy evaluation framework, in order to obtain a representative test dataset for verifying the effectiveness of our framework on evaluate the precision, recall, F-measure and the overall accuracy of query result, we use the toolkit of TPC_H to generate the test data. TPC_H is a toolkit provided by TPC which is an abbreviation of the Transaction Processing Performance Council; it is primarily used for OLAP test and to estimate the performance of business analysis in decision support systems; in addition, it contains a complete set of business-oriented ad-hoc queries and concurrent data modifications.

Firstly, we used the toolkit to generate the dataset, since the redundancy often exists in the real-world database. That is, there are usually more than one tuple describing one entity, so we then use one tuple as an entity and generate a tuple set whose number is randomly selected from 1 to 10, and meanwhile manually added errors to tuples in the set. In the case of synthetic labeled, we use the small data set which the tuples' number is 1K and 5K to evaluate the queries and the overall performance; we test the performance of data set's absolute accuracy whose data size is 10K, 20K, 30K, 40K and 50K, respectively; we also use large datasets to perform the efficiency experiments, whose data sizes are 20K, 40K, 60K, 80K and 100K, respectively.

For performance experiments, the precision, recall, F-measure and the global accuracy of query results are used as our evaluation criteria. For efficiency experiments, we use the ratio of the evaluation time and the actual execution time as the evaluation metric. When the operation is only related to the attributes of one dataset, the rough accuracy would be used to compare with the F-measure of the query to show the performance of the query. The others will be used to compare with the accuracy of the query results. To facilitate the description of the experimental results, we firstly summarize the main notations that will be used in the experimental part in Table 1.

**Table 1:** Main Notation.

Notation	Meaning
P-Actual	Actual precision
P-Evaluate	Estimated precision
R-Actual	Actual Recall
R-Evaluate	Estimated recall
F-Actual	Actual F-Measure
F-Evaluate	Estimated F-Measure
G-Accuracy	The accuracy of data source
Result Accuracy	The accuracy of query result
Offline Evaluation	The possible accuracy of query using the attribute accuracy calculated offline

### 6.2 The performance of absolute accuracy evaluation

**Figure 1:**
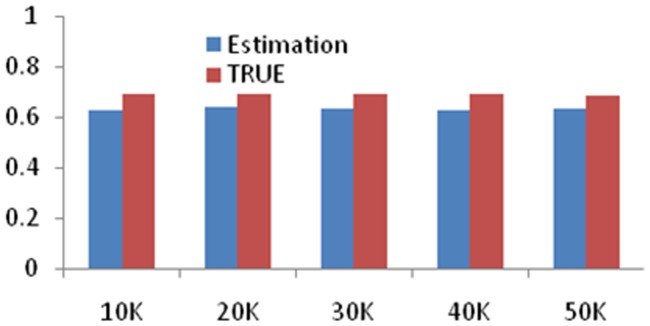
The experimental results of the comparison between the accuracy in presence and absence true values, denoted as True and Estimation, respectively. From the results, the evaluation of accuracy is littler than the true situation.

We use the small data set to test the performance of global accuracy evaluation, and formula (2).The data size is 10K, 20K, 30K, 40K and 50K, respectively. The results are shown in Figure 1.

As we can see from Figure 1, the evaluation of accuracy is a little lower than the true situation, but the deviation is little. Since the data came from one test instrument, the result is similar.

**Figure 2:**
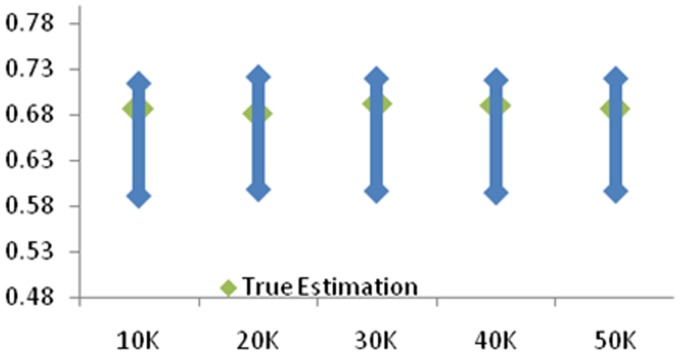
The experimental results of accuracy evaluation with functional dependencies. The range of the estimated accuracies and true values are shown as lines and dots, respectively. From the results, the true accuracy closes to the upper range. (**a**) 1K Selection (**b**) 5K Selection.

In order to improve the accuracy evaluation, we take into account of functional dependencies between attributes, consider only candidate keys and attributes with high accuracy, and remove the attribute with low accuracy. The results are shown in Figure 2.

As Figure 2 shows, the accuracy evaluation show in range form, and the method is effect, since the attributes have been pruned. And the true accuracy closes to the upper range.

### 6.3 The performance of relative accuracy evaluation

As mentioned before, all queries can be defined and derived by selection, projection, union, difference and Cartesian product. We carry out experiments to test the performance of selection, attributes union, relations union, relation difference and natural join.

#### 6.3.1 Selection

**Figure 3:**
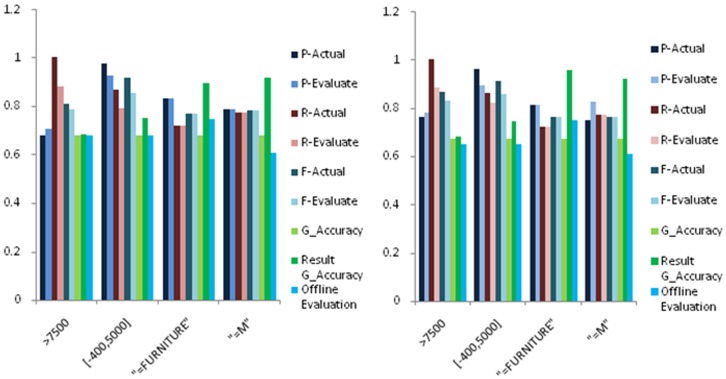
Experimental results for relative accuracy estimation of selection queries with different constraints, where we show P-Actual, P-Evaluate, R-Actual, R-Evaluate, G-Accuracy, Result Accuracy and Offline Evaluation with data size 1K and 5K. The meanings of these measures are shown in Table 1. (**a**) 1K Relation Union, Difference and Natural Join(**b**) 5K Relation Union, Difference and Natural Join. (**a**) 1K Attributes Union (**b**) 5K Attributes Union.

For selection, we perform experiments on three different attribute types independently. For the measurable types, the selection conditions include only one boundary and two boundaries; for comparable types, the selection conditions only include equivalent selection; for category types, the situation is the same as the comparable types. The results are shown in Figure 3.

As we can see from Figure 3, precision, recall and F-measure of the comparable and category type are very close to the true situation, and the error is within 10% of the exact evaluation; for the measurable type, as we use the mean value to represent the true value of entity. Sometimes it will appear large error ratio when the query boundary closed to the true value and the attribute itself with low accuracy, but the error is within 15% of the exact evaluation; as it is the operation between attributes, compared with actual F-measure, the offline estimation is slightly lower, but the error is within 15% of the actual estimation. In summary, our evaluation framework could give a good estimation for selection.

#### 6.3.2 Union

**Figure 4:**
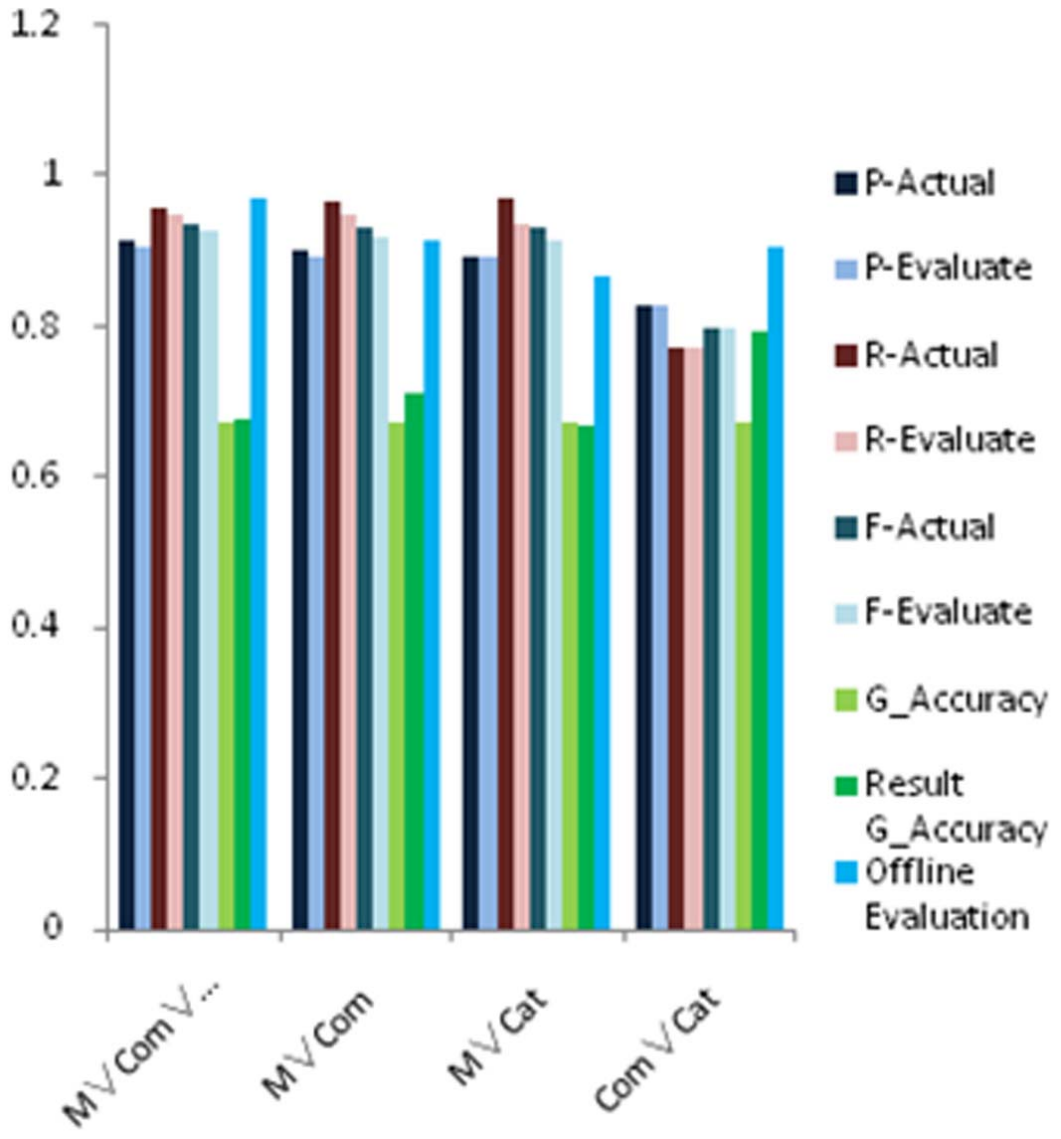
Experimental results for relative accuracy estimation of union queries with different sets, where we show P-Actual, P-Evaluate, R-Actual, R-Evaluate, G-Accuracy, Result Accuracy and Offline Evaluation with data size 1K and 5K. The meanings of these measures are shown in Table 1.

We first carry out experiments on attribute union, it belongs to selection 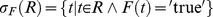, where 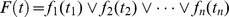. As there are three different attribute types, we tested all possible combinations of three types. The results are shown in Figure 4.

As observed from these figures, precision, recall and F-measure of attributes union are slightly lower than the true situation, but the error is within 5% of the exact values; as it is the operation between attributes, comparing with actual F-measure, the offline estimation is slightly higher. In practical applications, the offline estimation can be multiplied by a scaling factor which is less than 1 to improve the estimation accuracy of offline. As a conclusion, our evaluation framework could give a good estimation for relation union.

#### 6.3.3 Relation Union

**Figure 5:**
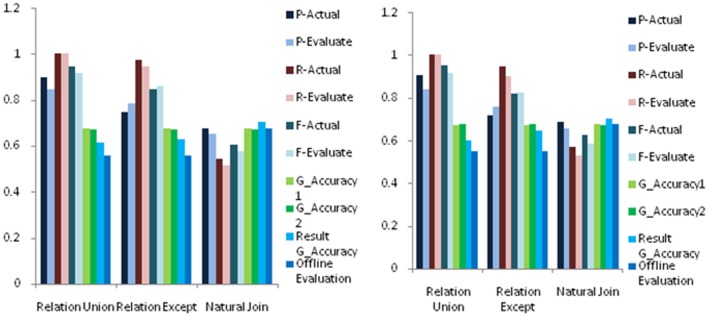
Experimental results for relative accuracy estimation of relational union, difference and natural join, where we show P-Actual, P-Evaluate, R-Actual, R-Evaluate, G-Accuracy 1, G-Accuracy 2, Result Accuracy and Offline Evaluation with data size 1K and 5K. The meanings of these measures are shown in Table 1 with G-Accuracy 1 and G-Accuracy 2 representing the accuracies of two input relations.

The relation union between dataset R and S is to find tuples which belong to R or S. The two datasets share some entities, but the errors added to the two datasets are independently. The results are shown in Figure 5.

As observed from these figures, precision, recall and F-measure are slightly lower than the true situation, but the error is within 5% of the exact values; as it is the operation between sets, compared with result's global accuracy, the offline estimation is slightly lower, but the error is within 10% of the estimation accuracy. To sum up, our evaluation framework could give a good estimation for relation union.

#### 6.3.4 Relation Difference

The relation difference between dataset *R* and *S* is to find tuples which belong to *R* but not S. For relations difference, the data set is same as relations union. The results are shown in Figure 5.

From these figures, precision, recall and F-measure fluctuate around the true situation, but the error is within 5% of the exact values; as it is the operation between sets, compared with result's global accuracy, the offline estimation is slightly lower, but the error is within 10% of the estimation accuracy. In summary, our evaluation framework could give a good estimation for difference.

#### 6.3.5 Natural Join

For Join, we only perform experiments on natural join, and others have the similar situations. The used attribute for join is comparable attribute. The results are shown in Figure 5.

From the experimental results, precision, recall and F-measure are slightly lower than the true situation, but the error is within 5% of the exact values; as it is the operation between sets, compared with result's global accuracy, the offline estimation is slightly lower, but the error is within 5% of the estimation accuracy. In conclusion, our evaluation framework could give a good estimation for join.

### 6.4 The efficiency of relative accuracy evaluation

**Figure 6:**
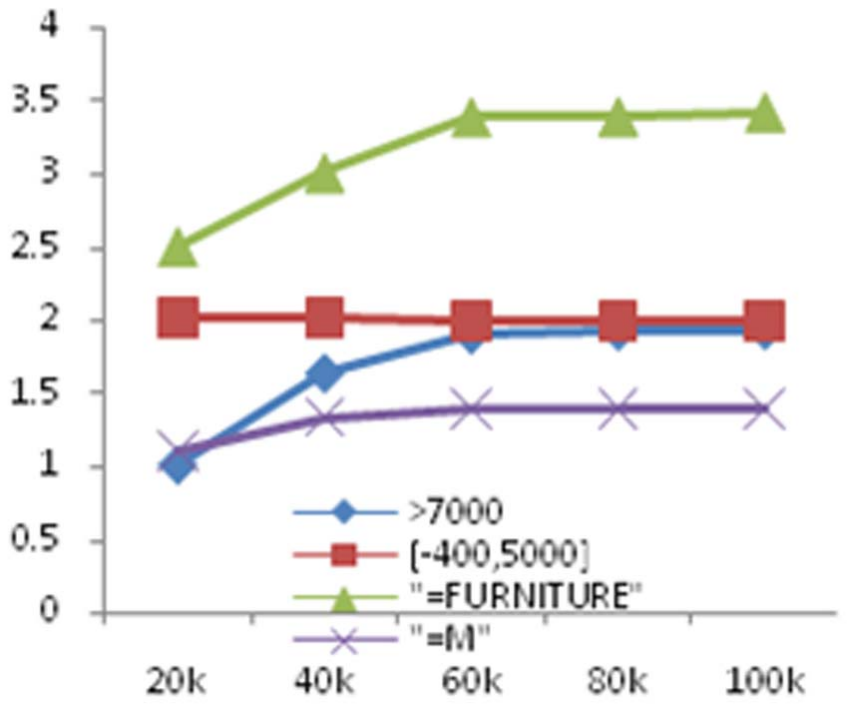
Experimental results on the scalability for accuracy estimation for selection queries with different constraints. The data size range from 20k to 100k and the unit of run time (*y*-axis) is second (s).

**Figure 7:**
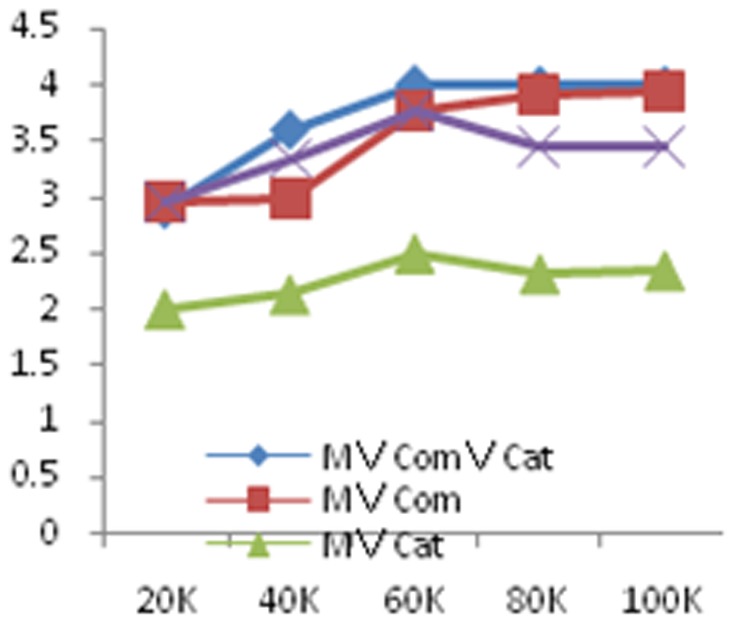
Experimental results on the scalability for accuracy estimation for attribute union queries with different sets. The data size range from 20k to 100k and the unit of run time (*y*-axis) is second (s).

**Figure 8:**
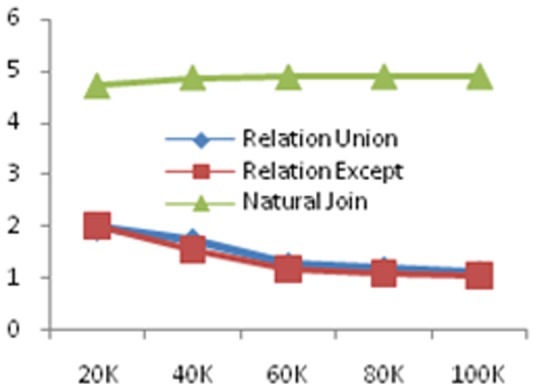
Experimental results on the scalability for accuracy estimation for relation union, except queries and natural join. The data size range from 20k to 100k and the unit of run time (*y*-axis) is second (s).

In order to test the efficiency of our framework, we execute experiments on different data sets with sizes 20k,40k,60k, 80k and 100k, respectively. We use the ratio of the evaluation time and the actual execution time as the evaluation metric and perform experiments on selection, attributes union, relations union, relations difference and natural join. The results are shown in Figure 6, Figure 7 and Figure 8, respectively.

From these figures, with the growth in the amount of data, for single attribute selection, the ratio of measurable attribute stable in 2 nearby; the ratio of comparable attribute stable in 3.5 nearby; the ratio of category attribute stable in 1.3 nearby. As the comparable attributes' calculation is related to the calculation of the edit distance, so that it takes a long time. For attributes union, the ratio is also stabilized in a constant with the increase in the amount of data; for relations union and relations difference, the ratio stable is in 1.2 nearby; for natural join, the ratio stable is in 5 nearby, this is mainly because the attributes' number of the result set is larger than the former relations. Since our framework evaluates not only the precision and recall of the query, but also the query result's global accuracy, the ration is larger than 1.As a summary, with the amount of data increases, our estimation framework can achieve linear time.

**Conclusion**: We carry out extensive performance and efficiency experiments on selection, attributes union, relations union, relations difference and natural join. For those queries, our evaluation methods could give accuracy estimation which is very close to the accuracy of the true situation, and for large amount of data, our algorithm can achieve linear time.

## Related Work

There are two classes of work related to our research, truth discovery and query evaluation. There are several studies related to the truth discovery. Resolving inconsistency [21] and modeling source quality [22] have been discussed in the context of data integration. Later [14] was the first to formally introduce the truth-finding problem. Then [23] developed several new algorithms and applied integer programming to enforce constraints on truth data [24]; designed a framework that can incorporate background information [25]; proposed an EM algorithm for truth finding in sensor networks. The copying relationship between sources was studied in [15]. But we consider the truth discovery from the point the entity recognition technology which was different from the previous works.

For query evaluation, many studies have focused on providing approximate answers to queries, but these techniques approximate query results based only upon a subset of data. In [26], Vrbsky et. al. studied how to provide approximate answers to set-valued queries. Other techniques use pre-computation [27], sampling [28] and synopses [29] to produce statistical results. Koch and Gotz [30] study the reliability of query results, but their goal is to provide a compositional framework for queries over unreliable data resulted from approximate query processing; Perez et al. study the evaluation of probabilistic threshold queries in MCDB [31]. But not as the precious work, our paper considers not only the relative accuracy of the query, but also the overall accuracy of query results.

## Conclusions

In this paper, we study the quality of the queries and design a relative accuracy evaluation framework for multi-modal data. Within this framework, we classify data types into three categories and develop accuracy evaluation algorithms for each category in cases of in presence and absence of true values. We present novel metric ARE for measuring the accuracy of one entity in statistic way, and also show the methods to evaluate the precision and recall of the basic queries, which would be used to combine with the absolute accuracy of query results to show the result's relative accuracy. Our framework could be easily extended to the big data, as we use the entity resolution technology as the foundation. We also propose the method to handle data update and to improve accuracy evaluation using functional dependencies. Extensive experimental results show the effectiveness and efficiency of our proposed framework.

As future work, we plan to combine the quality and copy relationship of data sourcesto improve the effectiveness of our framework.

## References

[1] Maykranz D, Seyfarth A (2014) Compliant ankle function results inlanding-takeoff asymmetry in legged locomotion. Journal of Theoretical Biology 349: 44–49. 24486249 10.1016/j.jtbi.2014.01.029

[2] Wang Z, Kokubo S, Tanimoto J, Fukuda E, Shigaki K (2013) Insight into the so-called spatial reciprocity. PHYSICAL REVIEW E 88: 042145. 10.1103/PhysRevE.88.042145 24229153

[3] Wang Z, Xia C, Meloni S, Zhou C, Moreno Y (2013) Impact of Social Punishment on Cooperative Behavior in Complex Networks. Sci Rep 3: 3055. 24162105 10.1038/srep03055 PMC3808815

[4] Wang Z, Szolnoki A, Perc M (2013) Interdependent network reciprocity in evolutionary games. Sci Rep 3: 1183. 23378915 10.1038/srep01183 PMC3560361

[5] Batini B, Scannapieco M (2006) Data quality: concepts, methodologies and techniques. Springer

[6] Raman D, Ton Z (2001) Execution: The missing link in retail operations. California Management Review 43 3: 136–152.

[7] English L (2000) Information quality management: The next frontier. DM Review Magazine

[8] Zhang Y, Wang H (2014) Accuracy Evaluation for Sensed Data. The Proceedings of WASA 2014: 205–214.

[9] Perc M (2013) Self-organization of progress across the century of physics. Sci Rep 3: 1720.

[10] Petersen AM, Tenenbaum JN, Havlin S, Stanley HE, Perc M (2012) Languages cool as they expand: Allometric scaling and the decreasing need for new words. Sci Rep 2: 943. 23230508 10.1038/srep00943 PMC3517984

[11] Perc M (2012) Evolution of the most common English words and phrases over the centuries. J. R. Soc. Interface 9: 3323–3328. 22832364 10.1098/rsif.2012.0491 PMC3481586

[12] Evans JA, Foster JG (2011) Metaknowledge. Science 331: 721. 21311014 10.1126/science.1201765

[13] Zhao B, Rubinstein BIP, Gemmell J, Han JW (2012) A bayesian approach to discovering truth from conficting sources for data integration. PVLDB 5 6: 550–561.

[14] Yin X, Han J, Yu PS (2007) Truth discovery with multiple conflicting information providers on the web. Proceeding of the KDD 1048–1052.

[15] Dong X, Berti-Equille L, Hu D, Srivastava D (2010) Solomon: Seeking the truth via copying detection. PVLDB 3 2: 1617–1620.

[16] Dong X, Berti-Equille L, Srivastava D (2009) Truth discovery and copying detection in a dynamic world. PVLDB 2 1: 562–573.

[17] Getoor L, Machanavajjhala A (2012) Entity Resolution: Theory, Practice and Open Challenges. PVLDB 5 12: 2018–2019.

[18] Al-ward YF (2010) Automatic Discovery of Candidate in the Relational Databases Keys by using Attributes Sets Closure. Journal of Al-Nahrain University Vol. 13 2: 247–255.

[19] Vilarem C (2002) Approximate key and foreign key discovery in relational databases. PHD Thesis, University of Toronto.

[20] Dempster AP, Laird NM, Rubin DB (1977) Maximum Likelihood from Incomplete Data via the EM Algorithm. Journal of the Royal Statistical Society 39 1: 1–38.

[21] Arenas M, Bertossi LE, Chomicki J (1999) Consistent query answers in inconsistent databases. Proceeding of the PODS 68–79.

[22] Florescu D, Koller D, Levy AY (1997) Using probabilistic information in data integration. Proceeding of the VLDB 216–225.

[23] Pasternack J, Roth D (2010) Knowing what to believe (when you already know something). Proceeding of the Internal Conference on Computational Linguistics 877–885.

[24] Pasternack J, Roth D (2011) Making better informed trust decisions with generalized fact-finding. Proceeding of theTwenty-Second International Joint Conference on Artificial Intelligence 2324–2329.

[25] Wang D, Abdelzaher T, Kaplan L, Aggarwal C (2011) On quantifying the accuracy of maximum likelihood estimation of participant reliability in social sensing. Proceeding of theDMSN 7–12.

[26] Vrbsky SV, Liu JWS (1994) Producing approximate answers to set- and single-valued queries. Journal of Systems and Software 27 3: 243–251.

[27] Poosala V, Ganti V (1999) Fast approximate query answering using precomputed statistics. Proceeding of the 15th ICDE 252.

[28] Gibbons PB, Matias Y (1998) New sampling-based summary statistics for improving approximate query answers. Proceeding of the 1998 ACM SIGMOD international conference on management of data 331–342.

[29] Acharya S, Gibbons PB, Poosala V, Ramaswamy S (1999) Join synopses for approximate query answering. Proceeding of the 1999 ACM SIGMOD international conference on management of data 275–286.

[30] Gotz M, Koch C (2009) A Compositional Framework for Complex Queries over Uncertain Data. Proceedings of the 12th International Conference on Database Theory 149–161.

[31] Perez L, Arumugam S, Jermaine C (2010) Evaluation of Probabilistic Threshold Queries in MCDB. Proceeding of the 2010 ACM SIGMOD international conference on Management of data 687–698.

